# Signal-to-noise and spatial resolution in in-line imaging. 1. Basic theory, numerical simulations and planar experimental images

**DOI:** 10.1107/S1600577524003886

**Published:** 2024-06-06

**Authors:** Timur E. Gureyev, David M. Paganin, Harry M. Quiney

**Affiliations:** a School of Physics University of Melbourne Parkville Victoria3010 Australia; b School of Physics and Astronomy Monash University Clayton Victoria3800 Australia; Australian Synchrotron, Australia

**Keywords:** X-ray imaging, computed tomography, phase contrast, spatial resolution

## Abstract

Signal-to-noise ratio and spatial resolution are quantitatively analysed in the context of in-line (propagation based) X-ray phase-contrast imaging. It is known that free-space propagation of a coherent X-ray beam from the imaged object to the detector plane, followed by phase retrieval in accordance with Paganin’s method, can increase the signal-to-noise in the resultant images without deteriorating the spatial resolution. This results in violation of the noise-resolution uncertainty principle and demonstrates ‘unreasonable’ effectiveness of the method. On the other hand, when the process of free-space propagation is performed in software, using the detected intensity distribution in the object plane, it cannot reproduce the same effectiveness, due to the amplification of photon shot noise. Here, it is shown that the performance of Paganin’s method is determined by just two dimensionless parameters: the Fresnel number and the ratio of the real decrement to the imaginary part of the refractive index of the imaged object. The relevant theoretical analysis is performed first, followed by computer simulations and then by a brief test using experimental images collected at a synchrotron beamline. More extensive experimental tests will be presented in the second part of this paper.

## Abstract 2

Signal-to-noise ratio and spatial resolution are quantitatively analysed in the context of propagation based X-ray phase-contrast imaging. The performance of the method is shown to be determined by the Fresnel number and the ratio of the real decrement to the imaginary part of the refractive index of the imaged object.

## 1. Introduction

X-ray phase-contrast imaging (PCI) is a class of powerful techniques for analysing the internal structure of non-crystalline samples (Paganin, 2006 ▸; Wilkins *et al.*, 2014 ▸; Endrizzi, 2018 ▸; Quenot *et al.*, 2022 ▸). Seminal results in this field were obtained in the 1990s, using coherent beams generated by synchrotrons (Snigirev *et al.*, 1995 ▸; Momose, 1995 ▸) and laboratory sources (Ingal & Beliaevskaya, 1995 ▸; Davis *et al.*, 1995 ▸; Wilkins *et al.*, 1996 ▸), although some closely related experiments were demonstrated earlier (Bonse & Hart, 1965 ▸; Ando & Hosoya, 1972 ▸; Förster *et al.*, 1980 ▸). X-ray PCI has a significant potential for increasing image contrast of biological soft tissues, compared with conventional absorption-based X-ray imaging. Various forms of X-ray PCI of biological samples have been demonstrated over the years (Wilkins *et al.*, 2014 ▸; Taba *et al.*, 2019 ▸; Endrizzi, 2018 ▸). In this work, however, we focus on just one method, known as in-line or propagation-based imaging (PBI) (Snigirev *et al.*, 1995 ▸; Wilkins *et al.*, 1996 ▸; Nugent *et al.*, 1996 ▸; Cloetens *et al.*, 1996 ▸). For a detailed history of the development of X-ray PCI technology and the current state of the art, see, for example, Paganin (2006 ▸), Wilkins *et al.* (2014 ▸), Endrizzi (2018 ▸) and Quenot *et al.* (2022 ▸).

PBI represents the simplest X-ray phase-contrast imaging technique, at least in principle. It does not require any optical elements in order to render phase contrast visible, relying instead on the free-space propagation of the beam transmitted through the sample before it is registered by a position-sensitive detector. The key requirement for PBI is a sufficiently high degree of spatial coherence of the illuminating beam (Wilkins *et al.*, 1996 ▸; Cloetens *et al.*, 1996 ▸; Paganin, 2006 ▸), which is typically achieved by using either highly collimated synchrotron radiation or micro-focus X-ray sources.

X-ray PCI was developed as a quantitative technique almost from the start, due to the use of associated phase retrieval methods (Momose, 1995 ▸; Nugent *et al.*, 1996 ▸; Gureyev & Wilkins, 1997 ▸; Paganin, 2006 ▸). This allowed, in particular, for development of phase-contrast computed tomography (PCT) (Momose, 1995 ▸; Raven *et al.*, 1996 ▸; Cloetens *et al.*, 1997 ▸). In the context of X-ray PBI, the most successful and widespread phase retrieval technique is Paganin’s method (Paganin *et al.*, 2002 ▸; Paganin, 2006 ▸), which is based on the homogeneous variant of the Transport of Intensity Equation (TIE) (Teague, 1983 ▸; Nugent *et al.*, 1996 ▸; Paganin *et al.*, 2002 ▸). Even though this method enables recovery of the phase from the registered intensity distribution in a single transverse plane, its main strength and the key reason for its popularity is its ability to significantly increase the signal-to-noise ratio (SNR) in an image, without deteriorating the spatial resolution (Paganin *et al.*, 2002 ▸; Gureyev *et al.*, 2014 ▸; Nesterets & Gureyev, 2014 ▸; Kitchen *et al.*, 2017 ▸). Although it may not be immediately obvious, the latter property is quite remarkable and even counter-intuitive, in view of the generic noise-resolution uncertainty (NRU) principle (Gureyev *et al.*, 2014 ▸, 2016 ▸, 2020 ▸; De Hoog *et al.*, 2014 ▸). Consider an imaging setup with a certain illumination area and a fixed total number of photons, which can be determined, for example, by the object to be imaged and the allowed radiation dose. The NRU is a generalization of the simple observation that a given allocation of photons can be either distributed in a smaller number of larger detector pixels, creating high SNR but low resolution, or in a larger number of smaller pixels, leading to higher resolution, but lower SNR. This trade-off has been shown to be quite fundamental: it is closely related to Shannon’s information capacity in imaging and can even be used to refine the Heisenberg Uncertainty Principle in some cases (Sakurai, 1967 ▸; Gureyev *et al.*, 2015 ▸, 2020 ▸). It was shown only relatively recently (Gureyev *et al.*, 2017*a* ▸) that the NRU is actually violated in Paganin’s method, not at the ‘phase retrieval’ stage but rather during the forward free-space propagation of the X-ray beam from the imaged object to the detector. The NRU states that the ratio of SNR to resolution cannot exceed the total number of photons divided by the imaged area. However, this is only true with respect to statistically independent photons, and the statistical independence can increase, in the sense that intensity correlations can decrease, upon free-space propagation. This fact is demonstrated, for example, in the van Cittert–Zernike theorem for intensity correlations (Goodman, 2000 ▸; Gureyev *et al.*, 2017b ▸). From the point of view of classical optics, it is easy to appreciate that the effective (angular) size of an incoherent X-ray source, which typically determines the degree of spatial coherence of an X-ray beam, becomes smaller with the increase of the propagation distance. The quantitative behaviour of the SNR and spatial resolution in synchrotron-based PBI experiments, and the details of the mechanism of the (beneficial) violation of the NRU in these experiments, is the main subject of the present study.

The physics of Paganin’s method can be understood on the basis of general signal transmission theory. As a wavefield propagates from the exit surface of a sample to the detector entrance surface, free-space diffraction amplifies the high-spatial-frequency part of the signal. This PBI ‘encoding’ step occurs before the addition of noise at the detector plane and the subsequent phase-retrieval ‘decoding’ step. Such a process can be viewed as a particular instance of signal transmission through a noisy channel (MacKay, 2003 ▸), for which the ‘noisy-channel coding theorem’ (Shannon, 1948*a* ▸,*b* ▸; MacKay, 2003 ▸) opens the logical possibility that an indirect three-step strategy – signal encoding, transmission through the noisy channel, signal decoding – may lead to SNR improvement without an increase in the total power of the signal or a decrease in its bandwidth*.* Letting **C** be the invertible operator that denotes the coding process, **N**_u_ be the operator via which uncorrelated noise is added to each pixel after the coding process, and **N**_c_ be the correlated-noise-addition operator induced by reversing the order in which the coding and noise addition are performed, **N**_c_ by definition obeys **N**_u_**C** = **CN**_c_, thus the similarity transformation **N**_c_ = **C**^−1^**N**_u_**C** maps uncorrelated to correlated noise. This transformation leaves the total power of the signal and its bandwidth unchanged. However, when the phase-retrieval decoding step (**C**^−1^) is applied to the noise in the detected image, uncorrelated (delta correlated) noise becomes correlated, decreasing the noise variance and leading to the beneficial violation of the NRU mentioned near the end of the previous paragraph.

A clarifying analogy is the Dolby noise reduction system introduced originally for compact music cassettes (Dolby, 1968 ▸). In Dolby noise reduction, the high temporal frequencies of an audio signal are amplified relative to their true value, before being transmitted through the noisy channel associated with recording onto magnetic audio tape, and then decoded by having the high temporal frequencies suppressed during playback via the Dolby filter. Crucially, this SNR-boosting process makes use of the prior knowledge that the high-frequency part of the target power spectrum decays more rapidly than the noise power spectrum, together with the assumption (for the purposes of our analogy) that the total power remains unchanged by the coding process. Similarly in PBI, the high-spatial-frequency components of a transmitted attenuated intensity map are amplified (in a total-power-preserving manner) relative to their true values. This amplification is enabled by the unitary-operator physics of paraxial free-space propagation from the exit surface of the sample to the entrance of the detector. The signal is then ‘transmitted’ through the continuous-to-discrete (Barrett & Myers, 2004 ▸) noisy channel of intensity registration via each pixel in the digital camera, and then decoded by having the high spatial frequencies suppressed during phase retrieval via the Paganin filter. This coding–transmission–decoding process results in a suppression of noise, without a loss of the high-spatial-frequency components, *i.e.* without a loss of spatial resolution.

We close this introduction with a brief overview of the remainder of the paper. Section 2 reviews some basics of in-line imaging using paraxial coherent scalar waves, including how to transition from the paraxial (wave) equation to the corresponding TIE and eikonal equations, together with a description of the form taken by the latter two equations for the case of plane waves passing through a homogeneous sample. Section 3 considers signal-to-noise ratio, spatial resolution and noise-resolution uncertainty in the context relevant to in-line imaging in both two and three spatial dimensions. The beneficial violation of the NRU in PBI is the topic of Section 4, with particular emphasis given to use of the homogeneous-sample version of the TIE in this setting. The key ideas of the preceding sections are illustrated with numerical and experimental examples, in Section 5. Section 6 contains concluding remarks.

## 2. In-line imaging

The propagation process of a scalar monochromatic electromagnetic beam in vacuum is described by the paraxial equation (Mandel & Wolf, 1995 ▸),
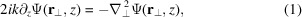
where 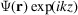 is the complex amplitude of the beam,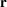 = 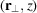 are Cartesian coordinates in the three-dimensional (3D) space, with *z* being the beam propagation direction and 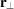 = 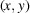 the position vector in transverse planes, 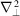 = 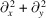 is the transverse two-dimensional (2D) Laplacian, *k* = 2π/λ is the wavenumber and λ is the wavelength. Equation (1) has the form of a 2D Schrödinger equation in free space, with *z* in the place of the time variable, the reduced Planck constant set to one and the mass replaced by the wavenumber. The paraxial equation represents the same kind of approximation to the Helmholtz equation as the Schrödinger equation does with respect to the Klein–Gordon equation (Sakurai, 1967 ▸). Substituting 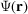 = 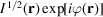, where *I*(**r**) is the intensity and φ(**r**) is the phase, into the Schrödinger or, more generally, into the Klein–Gordon equation leads to the de Broglie–Bohm formalism of quantum mechanics (pilot wave theory) (Bohm, 1952*a* ▸,*b* ▸; Nicolic, 2005 ▸). The same substitution in equation (1) leads to the following pair of equations for the intensity and phase of a monochromatic scalar electromagnetic beam (Teague, 1983 ▸),
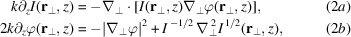
where 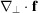 = 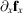 + 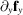 is the transverse divergence operator. Equation (2*a*) is the TIE; in the pilot-wave theory, it describes the propagation of particles along the field gradients. Equation (2*b*) is the eikonal (Hamilton–Jacobi) equation which, unlike the case of ray optics in free space, has an additional ‘diffraction’ term, 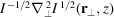. The diffraction term plays a role similar to the square of the refractive index in ray optics, both leading to bending of rays on propagation. In this sense, the diffraction term modifies the properties of space through which the rays propagate. In focal regions, the diffraction term also gives rise to the Gouy phase anomaly (Born & Wolf, 1999 ▸; Petersen *et al.*, 2014 ▸). In the pilot-wave theory, the diffraction term is associated with the ‘quantum potential’, which is responsible for the reciprocal effect of particles onto the field and makes the theory non-local in nature.

A complex amplitude 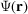 = 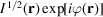 is called ‘monomorphous’ if the ratio of the phase and the logarithm of intensity, 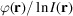, is the same at any position **r** (Paganin *et al.*, 2002 ▸). Monomorphous amplitudes arise, for example, in the object plane after transmission of an incident plane monochromatic X-ray wave through a ‘homogeneous’ object, *i.e.* an object consisting predominantly of a single material (Paganin *et al.*, 2002 ▸). Let *n*(**r**) = 1 − δ(**r**) + *i*β(**r**) denote the distribution of the complex refractive index inside such an object, where we omit dependence of all quantities on the wavelength for brevity. If an object consists of a single material, possibly with spatially varying density, the ratio γ(**r**) ≡ δ(**r**)/β(**r**) of the real decrement to the imaginary part of the refractive index has the same value at any point **r** inside the object (Paganin, 2006 ▸). The phase and the logarithm of intensity of a transmitted X-ray wave can typically be expressed as line integrals, 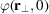 = 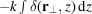 and 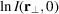 = 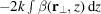, respectively, where we implicitly assume a plane monochromatic incident wave with unit amplitude (Paganin, 2006 ▸). It then follows that for a homogeneous object the transmitted complex amplitude is monomorphous with 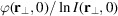 = 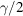. For such monomorphous complex amplitudes equations (2*a*) and (2*b*) decouple,
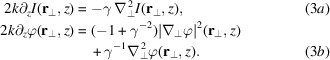
When γ 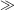 1, as is typically the case in hard X-ray PBI, equation (3*b*) has the form of a perturbation of the ray-optical eikonal equation (which formally corresponds to the case γ = ∞). The perturbation leads to weak bending of ray trajectories, which, in the first order of the small parameter γ^−1^, is proportional to the curvature of the wavefront.

In the present work, we are mostly interested in the linear finite-difference approximation to equation (3*a*), 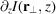

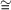

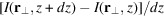
, which corresponds to sufficiently short propagation distances *dz* and converts equation (3*a*) into the so-called homogeneous finite-difference TIE (TIE-Hom) (Paganin *et al.*, 2002 ▸),
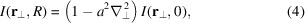
where *a*^2^ = γ*R*λ/(4π). Equation (4) provides a good approximation for the intensity distribution in in-line (propagation-based) images of monomorphous objects, if the object-plane intensity varies sufficiently slowly, so that 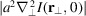

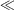

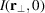
 (Paganin *et al.*, 2002 ▸).

## 3. Signal-to-noise ratio, spatial resolution and noise-resolution uncertainty

Consider first a very simple imaging system, in which a photon fluence, *S*_in_(**r**), radiated from a distant source and possibly scattered by an imaged object, is incident on a position-sensitive detector. The fluence is assumed to be expressed as the number of photons per area in 2D or per volume in 3D. The former case corresponds to conventional 2D images, while the latter case may correspond, for example, to computed tomography (CT), where the 3D ‘images’ can be the result of processing of the 2D projection images collected at different rotational positions of the imaged object.

It will be convenient to define the SNR and the spatial resolution via general expressions which are valid in any *n*-dimensional space. In particular, the SNR is defined as
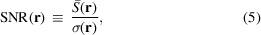
where 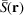 is the mean and 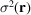 = 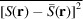 is the variance of the fluence *S*(**r**), with the overhead bar denoting the statistical average.

The spatial resolution can be expressed in terms of the width, defined via the second spatial integral moment, of the point-spread function (PSF), *P*(**r**),
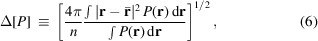
where 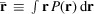. Note that 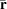 = 0 in the case of symmetrical PSFs. For non-negative functions *P*(**r**), 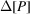 = 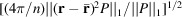, where ||*P*||_1_ is the first integral norm, corresponding to *n* = 1 in the expression 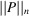

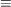

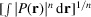
. In the following, all the considered PSFs will be non-negative and normalized, such that ||*P*||_1_ = 1, unless specifically mentioned.

One popular practical approach to measuring SNR and spatial resolution is based on the assumption of ‘local spatial ergodicity’ of the intensity distribution in an image. The latter means that, in a flat region of an image, where the spatial variation of intensity can be attributed to noise only (*i.e.* where the signal 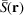 is approximately constant), a set of intensity measurements in adjacent locations can be considered as a representative sample of the statistical ensemble of intensity values at a given point of the image. In this case, the statistical mean and variance of intensity at a point **r** can be evaluated via spatial integrals over a vicinity Ω of that point,
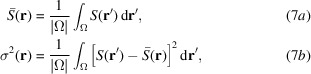
where |Ω| denotes the *n*-dimensional volume of Ω.

Note also the following parallel between the definitions of variance of a fluence and the spatial resolution expressed by equation (6). Let *H*_**r**_(*s*) be the probability distribution function (PDF) of fluence *S*(**r**). Then 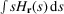 = 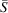 and 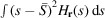 = 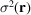, and hence σ^2^(**r**) = [1/(4π)]Δ^2^[*H*]. In other words, the variance of a fluence is proportional to the square of the width of its PDF, which is a rather straightforward observation. The link between the variance of image intensity and the spatial resolution is exploited in the NRU principle (Gureyev *et al.*, 2014 ▸, 2016 ▸; De Hoog *et al.*, 2014 ▸), which is described next. The NRU states that, for any function, its spatial width and the width of its PDF cannot be made arbitrarily small at the same time (Gureyev *et al.*, 2020 ▸). This accords with the simple idea that blurring a normalized image with a unit-strength low-pass filter will narrow the intensity’s PDF by reducing noise, at the cost of broadening the width of the PSF. For an intuitive pictorial representation of this key trade-off that underpins the NRU, we refer the reader to Fig. 1 of Gureyev *et al.* (2020 ▸).

As hinted at towards the end of the previous paragraph, the detected fluence, *S*_D_(**r**), can be often expressed in the form of a convolution, *S*_D_ = *S*_in_*D, of the fluence incident on the detector, *S*_in_(**r**), with the non-negative PSF of the detector, *D*(**r**),
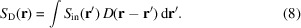
We assume for now that the incident fluence is almost uncorrelated and uniform over length scales comparable with the width of *D*(**r**). In other words, we assume that the correlation length, *h*, of the incident fluence is much smaller than the width of the PSF, while 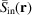 and 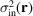 are both almost constant over distances comparable with the width of *D*(**r**). In that case, the effect of the detector PSF on the SNR can be described as follows (Gureyev *et al.*, 2016 ▸),
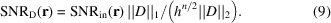
We note that the quantity
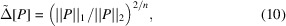
which appears in the right-hand side of equation (9), can be used as a measure of the width of the function *P*(**r**) (Mandel & Wolf, 1962 ▸; Gureyev *et al.*, 2016 ▸). When *P* represents the PSF of an imaging system, equation (10) provides an alternative definition of spatial resolution, which has a different mathematical form from equation (6), but often produces comparable results. For example, for Gaussian PSFs, 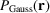 = 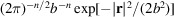, we obtain 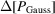 = 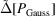 = 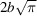 for any *n*. In the case of a top-hat function with width 2*b*, 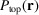 = 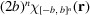, where 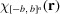 is equal to 1 inside the *n*-dimensional cube [−*b*, *b*]^*n*^ and is equal to 0 everywhere else, we get Δ[*P*_top_] = 2*b*(π/3)^1/2^ ≅ 2.05*b* and 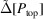 = 2*b*. If 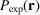 = 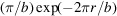 is an exponential distribution, then 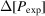 = 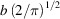 and 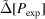 = 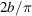. Note, however, that in the case of 1D Cauchy (Lorentzian) distributions, *P*_Cauchy_(*x*) = (*b*/π)/(*b*^2^ + *x*^2^), we get Δ[*P*_Cauchy_] = ∞, while 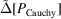 = 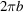.

Using the definition from equation (10), equation (9) can be re-written as
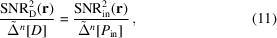
where 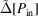 = *h*. Note that the noise correlation length, *h*, can be associated with the width of a function, *P*_in_, whose autocorrelation is equal to the degree of spatial coherence of the incident fluence (Gureyev *et al.*, 2016 ▸).

Now consider the case of linear filtering of the registered image, which can be described by the convolution *S*_D_*F,*i.e.* by equation (8) with the detected fluence *S*_D_(**r**) instead of the incident fluence and a non-negative filter function *F*(**r**) instead of the PSF *D*(**r**). According to the associativity and commutativity of the convolution operation, *S*_D_*F = (*S*_in_*D)*F = *S*_in_*(D*F) = *S*_in_*(F*D). Therefore, if (F*D)(**r**) is almost constant over distances of the order of *h*, but varies much faster than 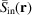 and 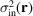, then, arguing exactly as above, we find that after such filtering the ratio of SNR^2^ to the effective ‘resolution volume’ 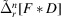 must remain unchanged (Gureyev *et al.*, 2016 ▸),
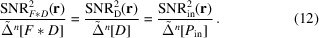
Equation (12) shows that the ratio of SNR^2^ to the corresponding resolution volume is constant in linear shift-invariant transformations. In the case of Poisson photon statistics, SNR^2^ is equal to the number of photons. Therefore, in this case equation (12) is just a restatement of the simple fact that larger effective voxels, created as a result of image filtering or binning, contain more registered photons, leading to the proportionally larger SNR^2^. Equation (12) can be alternatively understood as a statement that an increase in the photon correlation length leads to a proportional increase in the SNR, which is a well known effect of conventional low-pass filtering of images.

The trade-off between the SNR and spatial resolution is captured in a more general context by the NRU principle (Gureyev *et al.*, 2016 ▸, 2020 ▸) which states that, for a fixed photon fluence, any gain in the SNR is equal to or less than the corresponding increase of the minimal spatially resolvable volume. Mathematically, the NRU can be expressed as
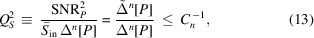
where *Q*_*S*_ is called the ‘intrinsic imaging quality’ characteristic and *C*_*n*_ is the Epanechnikov constant: *C*_1_ = (6/5)(π/5)^1/2^ ≅ 19/20,*C*_2_ = 8/9 and *C*_3_ = 60(π/7^5^)^1/2^ ≅ 4/5 (Gureyev *et al.*, 2014 ▸, 2016 ▸; De Hoog *et al.*, 2014 ▸). The upper limit (equal to 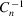) in equation (13) is achieved for Epanechnikov PSFs, *P*_Epan_(**r**) = *A*_*n*_(1 − |**r**|^2^/ *b*^2^)_+_, where *A*_*n*_ and *b* are constants, and the subscript ‘+’ means that all negative values inside the brackets are replaced by zero (De Hoog *et al.*, 2014 ▸). It follows from equation (13) that 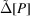

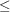

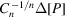
, *i.e.*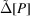 generally provides a more ‘optimistic’ estimate of the spatial resolution compared with Δ[*P*], which can be observed in the examples given above. Equation (13) can be extended to the cases of linear filtering of detected images, in the same way as equation (12) extends equation (11), showing, in particular, that the intrinsic imaging quality *Q*_*S*_ remains unchanged after linear filtering (such as, for example, convolution or deconvolution with a non-negative function) of images (Gureyev *et al.*, 2016 ▸).

A related result is represented by the mathematical form of the classical Heisenberg Uncertainty Principle (HUP) (Sakurai, 1967 ▸; Folland & Sitaram, 1997 ▸),
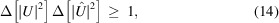
where *U*(**r**) is an arbitrary complex-valued square-integrable function and the overhead hat symbol denotes the Fourier transform, 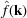 = 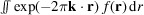. This inequality implies that the minimal phase-space volume is bounded from below for any square-integrable function. The lower limit in the HUP is equal to one and is achieved for Gaussian functions 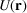 = 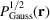, for which one obtains 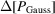 = 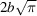 and
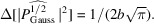
It has been shown (Gureyev *et al.*, 2015 ▸) that the NRU can be used to refine the HUP, replacing the right-hand side in equation (14) by the maximum of 1 and 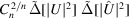. The latter functional can be either larger or smaller than one for different functions *U*(**r**) (Gureyev *et al.*, 2015 ▸).

We are going to apply the above results to measurements of SNR and spatial resolution in 2D and 3D images. Assuming that spatial ergodicity is satisfied in a sufficiently large area of the relevant images, we will use discrete analogues of equations (7*a*) and (7*b*), for estimation of the mean and variance of intensity in a pixel located in a flat area of the image, via the mean and variance calculated over a set of adjacent pixels. This will allow us to evaluate the SNR via equation (5). For estimations of the spatial resolution, we will use a method based on the Fourier transform of equation (8),
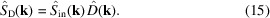
If, as assumed after equation (8), the noisy incident fluence is uncorrelated and is almost flat within a given region of the image, then the Fourier transform of the fluence is also a flat noisy distribution. Therefore, the width of the product of the two functions in the right-hand side of equation (15) is determined primarily by the width of the modulation transfer function (MTF), 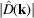. Assuming that the PSF is Gaussian, and hence the MTF is also Gaussian, we can use the known relationship between the widths of a Gaussian distribution and its Fourier transform to evaluate the width of the PSF from the measured width of the MTF,
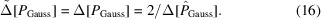
Note, however, that the relationship between the width of a function and the width of its Fourier transform, being always reciprocal in nature, does not have the same proportionality constant for all functions. In this respect, the relevant result is represented not by the HUP, equation (14), but by the Laue inequality (Dreier *et al.*, 2001 ▸),
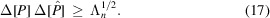
Equation (17) can only guarantee that the product of the width of a function and the width of its Fourier transform is always larger than a certain absolute constant. The maximum possible value on the right-hand side of equation (17) is called the Laue constant, and it is known to be in the range 0.54 < Λ_*n*_ < 0.85. Unlike the case of the NRU, equation (13), or the HUP, equation (14), neither the exact value of Λ_*n*_ nor the functional form of the ‘minimizer’, corresponding to a function *P*(**r**) for which the left-hand side of equation (17) [Disp-formula fd17] reaches its minimal possible value, are known. A method for measuring spatial resolution which is closely related to equations (15)–(17) was also developed and used previously (Mizutani *et al.*, 2016 ▸; Brombal *et al.*, 2018 ▸, 2019 ▸).

## 4. Violation of NRU in propagation-based imaging

Equations (11)–(13) demonstrate that NRU is satisfied in detection and linear filtering of images. On the other hand, it is known that propagation-based (also known as in-line) imaging, which is described by equation (4), exhibits an ‘unreasonable’ effectiveness and can violate the NRU principle (Gureyev *et al.*, 2017*a* ▸). This means that PBI can produce a gain in SNR without a loss of spatial resolution or improve the spatial resolution without a loss of SNR.

Consider the case of PBI of monomorphous complex wave amplitudes, as described by TIE-Hom. Equation (4) can be trivially re-written as a convolution,
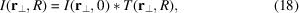
where 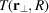 = 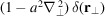 and 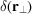 is the Dirac delta-function. It can be verified by direct calculations that the second integral moment of 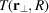 is equal to −4*a*^2^. The fact that this second integral moment is negative means that equation (18) acts as a deconvolution (Gureyev *et al.*, 2003 ▸, 2004 ▸, 2017*a* ▸), effectively improving the spatial resolution in PBI images, 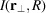, in comparison with the corresponding object-plane images, 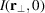.

In a real experiment, the detected intensity distribution in the object plane can often be represented as a convolution, 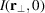 = 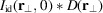. Here 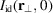 is the ‘ideal’ object-plane intensity distribution corresponding to a delta-function detector PSF and 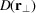 is the real detector PSF. In a more general setting, with incident illumination other than a plane wave, the image blurring can also include a contribution from the spatial distribution of the source intensity (Gureyev *et al.*, 2008 ▸). After the substitution 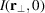 = 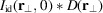, equation (18) becomes
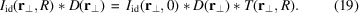
While the filter function 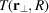 is singular, its convolution with 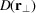 can be a smooth function. For example, in the case of a 2D Gaussian PSF, 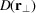 = 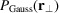, with variance equal to 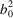 = 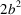, we obtain 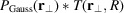 = 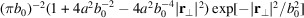, which is a smooth function. The second integral moment of this function is equal to 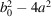, which can still be negative, in principle, if *b*_0_ < 2*a*. However, since the second moments of the left-hand and the right-hand sides of equation (19) must be equal, and the intensity distribution in the object plane is obviously non-negative, it implies that 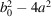

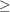

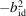
, where 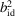 is the second integral moment of the function 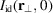.

The fact that the filter function 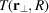 has negative as well as positive values may be presumed to be a reason why the NRU, which has been only proven for non-negative filter functions (De Hoog *et al.*, 2014 ▸), does not apply to it. Let us show, however, that in fact equation (18), and hence equation (4), still preserve the ratio of SNR^2^ to the spatial resolution volume.

Equation (4) has an exact inverse (Paganin *et al.*, 2002 ▸; Paganin, 2006 ▸), *e.g.* in the space of tempered distributions (Vladimirov, 2002 ▸),
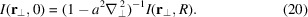
The inverse operator in the right-hand side of equation (20) can be expressed with the help of the Fourier transform,
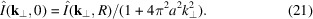
Equation (21) is known as the TIE-Hom retrieval equation or Paganin’s method (Paganin *et al.*, 2002 ▸). Due to its noise robustness and ease of practical application, it has been successfully employed in a large variety of phase-contrast imaging scenarios using different forms of radiation and matter waves. As particular examples of this noise robustness, dose reductions by a factor of thousands or more are achievable for in-line imaging in CT (Kitchen *et al.*, 2017 ▸), thereby enabling synchrotron-based X-ray PCT at the rate of 1000 tomograms per second (García-Moreno *et al.*, 2019 ▸, 2021 ▸).

Taking the inverse Fourier transform of equation (21), it is possible to re-write equation (20) in the form of a convolution,
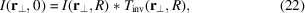
where 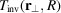

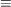

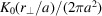
 is the inverse Fourier transform of the MTF 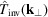 = 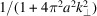 from equation (21) and *K*_0_ is the zero-order modified Bessel function of the second kind (Abramowitz & Stegun, 1972 ▸; Nesterets & Gureyev, 2014 ▸). The second integral moment of the TIE-Hom retrieval filter function, 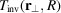, is equal to 4*a*^2^, and thus, as expected, it is equal to minus the second moment of 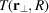. As the function *K*_0_(ρ) is positive for any positive ρ, the convolution with 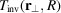 satisfies the NRU conditions. Accordingly, the transformation represented by equation (22) increases the SNR in the exact proportion to the deterioration of the spatial resolution, so that equation (12) holds for it (with *n* = 2). This allows us to conclude that, if equation (4) violated the NRU, *e.g.* increased SNR by a factor 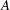

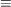

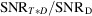

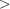

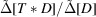
, then by applying equation (4) and its inverse, equation (20), in sequence it would have been possible to increase the SNR, while leaving the intensity distribution unchanged,
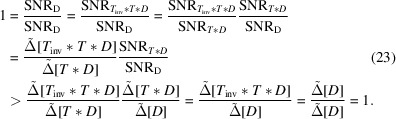
The fourth equality in equation (23) is the NRU applied to equation (22), while the subsequent inequality in equation (23) is the result of the substitution of the above assumption about the factor *A*. The obvious contradiction, 1 > 1, obtained in equation (23) as a result of the latter assumption, means that, in fact, the filter function 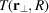 = 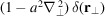 must obey the NRU, despite not being positive everywhere. The same logic can be used to prove that any filter function *F*(**r**), whose ‘inverse’ function, *F*_inv_(**r**), such that 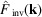

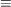

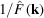
, exists (*e.g.* in the space of tempered distributions), is non-negative and satisfies the conditions for ‘well behaved’ point-spread functions described above, must also satisfy the NRU.

However, the fact that equation (4), which is typically used to describe PBI imaging of monomorphous objects (Paganin *et al.*, 2002 ▸), satisfies the NRU still does not actually prohibit the violation of NRU in PBI. Let us recall that equation (4) is valid only for sufficiently slowly varying intensity distributions. In PBI experiments, the average phase function, 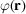 = 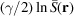, often varies sufficiently slowly for the latter condition to be satisfied and, hence, for equation (4) to be applicable. However, the noise component, 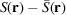, typically varies very rapidly from pixel to pixel. Therefore, the free-space propagation of the detected photon fluence, *S*(**r**), cannot be correctly described by equation (4), allowing for the possibility of violation of the NRU in PBI.

An equation generalizing equation (4) to rapidly varying functions is also known (Gureyev *et al.*, 2006 ▸),
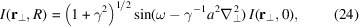
where 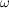

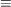

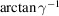
. It is not known to us if equation (24) has an inverse in the space of tempered distributions, which could be represented as a convolution with a positive function, as was the case with equation (22). The Fourier transform of the convolution kernel 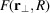 = 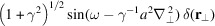 in equation (24) is equal to 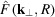 = 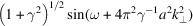. Unlike the case of equation (21), the latter function becomes zero at certain values of 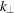. Thus, the arguments used above to prove that equation (4) satisfies the NRU may not apply to equation (24). On the other hand, an inverse of a function with isolated zero values may still belong to the space of tempered distributions and may be positive, in principle (Vladimirov, 2002 ▸). Nevertheless, as demonstrated by a numerical example in the next section, for some input functions 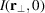, equation (24) amplifies noise in proportion to, or even stronger than, the corresponding gain in the spatial resolution. Therefore, in such cases, equation (24) also cannot explain the observed violation of the NRU in PBI.

A solution to the above ‘paradox’, which suggests an explanation for the violation of NRU in PBI, can be obtained in the following way. As shown by Gureyev *et al.* (2017*a* ▸), the violation of NRU in PBI can take place when the image noise is dominated by the shot noise of the photodetection process. In that case, since the paraxial free-space propagation preserves the number of photons, the SNR is the same in flat areas of the ‘contact’ object-plane and the propagated image-plane images. At the same time, the spatial resolution is improved upon free-space propagation, as explained after equation (18) above. Therefore, the SNR to resolution ratio is improved upon free-space propagation, thus seemingly violating the NRU. Subsequently, the SNR is improved upon the TIE-Hom retrieval, as this process corresponds to a low-pass filtering of the image fluence, according to equation (22). As equation (22) conforms to the NRU, the spatial resolution deteriorates after its application. If the TIE-Hom retrieval is performed with the ‘true’ value of the parameter *a*^2^ = γ*R*λ/(4π), it effectively inverts the effect of the forward free-space propagation, which is well approximated by equation (18). The spatial resolution is then returned to its original value in the object plane, while the SNR is increased in comparison with the image plane, the latter being equal to SNR in the ‘contact’ images in the object plane at the same incident fluence. Overall, after the free-space propagation, followed by the TIE-Hom retrieval, the SNR is increased compared with the ‘contact’ images of the same object at the same dose, while the spatial resolution remains unchanged. This qualitatively explains the mechanism behind the beneficial violation of the NRU in PBI. Now, let us study the corresponding phenomena quantitatively.

Consider first the effect of the TIE-Hom retrieval on the SNR (Nesterets & Gureyev, 2014 ▸). For simplicity, assume that the PSF in the image plane *z* = *R* is equal to the PSF of the detector, 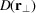, which is much narrower than the TIE-Hom retrieval filter function, *i.e.*
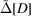

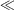

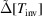
. In this case, the spatial resolution after the TIE-Hom retrieval is approximately equal to the width of the filter function: 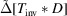

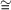

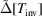
. It can also be verified directly that 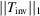 = 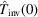 = 1, 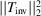 = 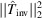 = 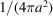 = 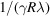 and hence 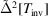 = 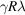. It then follows from the invariance of the SNR-to-resolution ratio, equation (12), that an application of equation (22) increases SNR by the factor equal to the ratio of the corresponding spatial resolutions, 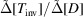,
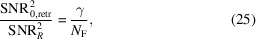
where 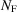

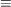

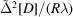
 is the Fresnel number equal to the square of the ratio of the detector resolution and the width of the first Fresnel zone (Gureyev *et al.*, 2009 ▸), SNR_0,retr_ is the SNR in the object plane, *z* = 0, after TIE-Hom retrieval and SNR_*R*_ is the SNR in the image plane, *z* = *R*. Since the number of photons is preserved in free-space propagation, we have 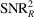 = 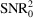, where SNR_0_ is the SNR in the contact images in the object plane. Equation (25) then implies
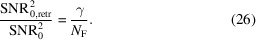
As mentioned above, the width of the PSF is increased upon the TIE-Hom retrieval by the same amount as it is reduced upon the free-space propagation. Therefore, after the free-space propagation followed by the TIE-Hom retrieval with *a*^2^ = γ*R*λ/(4π), the spatial resolution remains unchanged. In particular, Δ[*P*_0,retr_] = Δ[*P*_0_], where *P*_0,retr_ is the effective PSF after the free-space propagation followed by TIE-Hom retrieval and *P*_0_ is the original PSF in the object plane. Combining this with the increase in the SNR in accordance with equation (26) and dividing the ratios of the SNR to resolution by the square root of the corresponding incident fluence, we obtain the following expression for the ‘gain factor’ (Nesterets & Gureyev, 2014 ▸; Gureyev *et al.*, 2017*a* ▸),
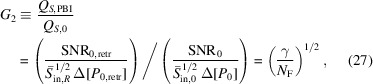
where *Q*_*S*,PBI_ and *Q*_*S*,0_ are the intrinsic quality characteristics of the PBI and ‘contact’ imaging, respectively, with 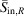 and 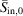 being the corresponding incident fluences. Note that it was implicitly assumed in equation (27) that SNR is proportional to the square root of the incident fluence, as is the case for Poisson statistics. Since, for any given object, the absorbed radiation dose is proportional to the incident fluence, it is also possible to replace the incident fluence by the dose in equation (27). We will use the latter fact in our analysis of experimental images below.

Recall that in the derivation of equation (25) we assumed that 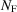 = 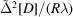

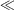

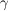
. Under such conditions, the gain factor *G*_2_ can be large. In a PBI experiment involving hard X-ray imaging of biological samples, γ can typically be of the order of 10^3^ and *N*_F_ can be of the order of 10. According to equation (27), this can lead to gain factors of the order of 10 in 2D. Furthermore, as shown by Nesterets & Gureyev (2014 ▸) (see also the second part of the present paper), the gain factor in 3D, *i.e.* in PBI CT, is equal to *G*_3_ ≅ γ/*N*_F_, which can be of the order of 10^2^. The latter values of the 3D gain factor correspond to dose reduction of the order of 10^4^, compared with conventional CT at the same dose, without any loss of spatial resolution – see details given by Kitchen *et al.* (2017 ▸) and in the second part of the present paper.

Another interesting feature of equation (27) is that the gain factor remains the same regardless of the value of γ′ that is used at the TIE-Hom retrieval stage of PBI. In other words, the gain factor remains the same if one chooses to apply the TIE-Hom retrieval operator 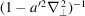 with *a*′^2^ = γ′*R*λ/(4π), where γ′ is different from the ‘true’ value of γ = δ/β appearing in equation (27). This invariance is a simple consequence of the fact, proved in Section 4 above, that the TIE-Hom retrieval operator 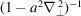 does not change the SNR to spatial resolution ratio, regardless of the value of the parameter *a*. The ‘magic’ of PBI imaging, which can lead to beneficial violation of the NRU and gain factors larger than one, happens at the forward free-space propagation stage of the process, while the subsequent ‘phase retrieval’ stage does not involve any ‘magic’, leaving the SNR-to-resolution ratio unchanged.

## 5. Numerical simulations and an experimental example

### 5.1. Numerical simulations of PBI imaging

**Figure 1:**
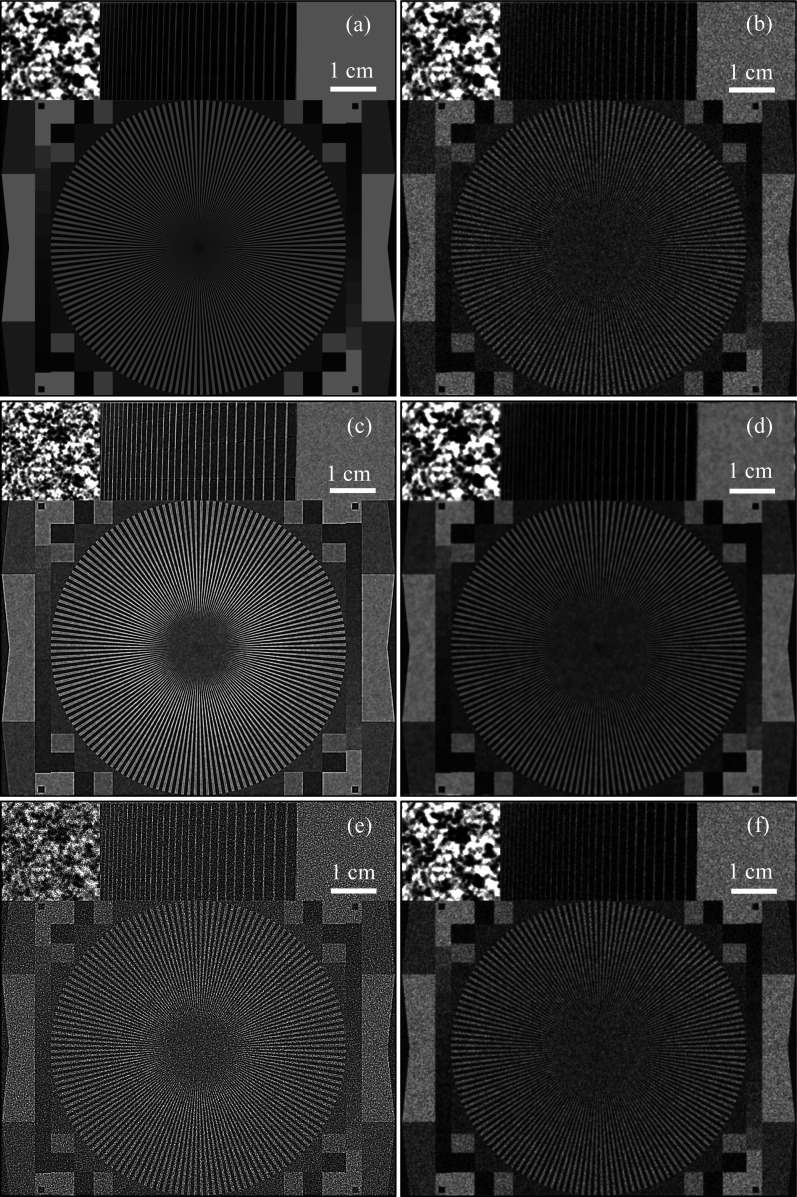
(*a*) Original transmission function, 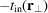. (*b*) Noisy blurred detected fluence in the object plane, 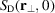. (*c*) Noisy blurred detected fluence in the image plane, 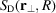. (*d*) Distribution 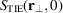, obtained by TIE-Hom phase retrieval from 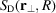. (*e*) Distribution 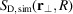, obtained by simulated free-space propagation of the homogeneous complex amplitude produced from 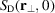. (*f*) Distribution 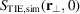, obtained by TIE-Hom retrieval from 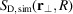.

Here we consider the case of a plane monochromatic incident X-ray wave 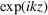, with *k* = 2π/λ and λ = 1 Å. The incident wave illuminated a thin homogeneous sample, with γ = 100 at the chosen wavelength. The sample was located immediately before the object plane *z* = 0. All images were assumed to be collected by an X-ray detector with a sensitive area of 10.24 cm × 10.24 cm occupied by 4096 × 4096 pixels. The size of the detector pixels was 25 µm × 25 µm. The X-ray transmission through the sample was modelled with the help of a function 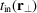, which had the values in the interval (0, 0.1) and was spatially distributed as in Fig. 1(*a*) ▸. The transmitted complex amplitude in the object plane was 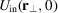 = 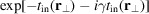. The distribution 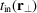 represented a low-pass filtered version of imaging test patterns, which contained features with different contrasts and details containing a wide range of spatial frequencies. Low-pass filtering, using a Gaussian convolution kernel with FWHM of 100 µm (4 pixels), was applied to the original test patterns to create the function 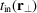. This was done in order to satisfy the requirement for the incident fluence to be slowly varying compared with the detector resolution. We inserted a square region with 1024 × 1024 pixels in the top right corner of the image, with 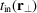 = 0 and, hence, 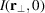 = 1, inside this region. This flat region was used for accurate evaluation of the SNR and spatial resolution. We also inserted another square region with 1024 × 1024 pixels in the top left corner of the image, this region containing a pseudo-random distribution which was obtained by applying Poisson noise with standard deviation equal to 0.1 to a uniform region of the same size, with 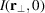 = 1, and then low-pass filtering the result with a Lorentzian filter with a FWHM of 1054 µm. The presence of this pattern in the image allowed us to quantitatively evaluate the improvement in the spatial resolution after the free-space propagation.

In order to simulate the photon shot noise in the detected intensity, we simulated pseudo-random Poisson noise with standard deviation equal to 20% of the average transmitted intensity, 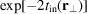. This corresponds to an average fluence of 25 photons per pixel [since 1/(25)^1/2^ = 0.2]. We subsequently convolved the noisy fluence with the detector PSF, 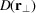, which was modelled as a 2D circular Gaussian distribution with σ = 125 µm. The resultant noisy blurred detected fluence, 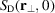, is shown in Fig. 1 ▸(*b*). We also calculated the free-space propagation of the complex amplitude 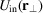 from the object plane *z* = 0 to the image plane *z* = *R* = 100 m by evaluating the corresponding Fresnel diffraction integrals. We simulated 20% Poisson noise in the image-plane fluence, as in the object plane, before convolving the noisy fluence with the same detector PSF as in the object plane. The resultant noisy blurred detected fluence in the image plane, 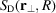, is shown in Fig. 1 ▸(*c*). We then applied the TIE-Hom phase retrieval, equation (20), to 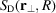, with the result, 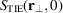, shown in Fig. 1 ▸(*d*). We also calculated the free-space propagation from the object plane *z* = 0 to the image plane *z* = 100 m of the complex amplitude 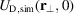 = 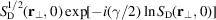, produced from the noisy blurred registered fluence in the detector plane. The resultant intensity distribution in the image plane, 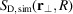, can be seen in Fig. 1 ▸(*e*). Finally, we applied the TIE-Hom phase retrieval, equation (20), to 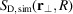, with the result, 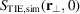, shown in Fig. 1 ▸(*f*). Since parameters of this simulation included a Gaussian detector PSF with *b* = 125 µm, the propagation distance *R* = 100 m and the X-ray wavelength λ = 0.1 Å, the corresponding (minimal) Fresnel number was *N*_F_ = 4π*b*^2^/(*R*λ) ≅ 19.6.

Examining Fig. 1 ▸(*c*), one can notice that, on a qualitative level, the forward propagation of the complex amplitude sharpened the image, without increasing noise. The improvement in the spatial resolution can be observed by comparing the following features of Figs. 1 ▸(*b*) and 1 ▸(*c*). Firstly, the convergent straight lines in the circular ‘star’ pattern can be discerned closer to the centre of the pattern in Fig. 1 ▸(*c*), compared with Fig. 1 ▸(*b*). This is a known manifestation of a visual effect of improved spatial resolution for which such ‘star’ patterns are included in various image resolution standards. Secondly, the sub-image in the top-left corner shows noticeably higher spatial frequencies in Fig. 1 ▸(*c*), compared with Fig. 1 ▸(*b*). Finally, one may also notice that the straight horizontal and vertical edges between different image components in Fig. 1 ▸(*c*) are sharper than in Fig. 1 ▸(*b*). The noise level in the two images is the same by construction: the same level of Poisson noise was added to both images before applying the same Gaussian detector PSF with a standard deviation of 5 pixels. The image in Fig. 1 ▸(*c*) is substantially less noisy than that in Fig. 1 ▸(*e*), which contains the result of numerical free-space propagation of a complex amplitude created from the noisy detected fluence in Fig. 1 ▸(*b*) using the homogeneous complex amplitude 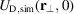. As a consequence, after the application of TIE-Hom retrieval to Fig. 1 ▸(*c*), the result in Fig. 1 ▸(*d*) looks less noisy than Fig. 1 ▸(*f*), which contains the result of application of TIE-Hom retrieval to Fig. 1 ▸(*e*). The fact that Fig. 1 ▸(*d*) is also less noisy than the object-plane intensity distribution in Fig. 1 ▸(*b*), while being as sharp as the latter, is consistent with the ‘unreasonable’ effectiveness of PBI imaging (Gureyev *et al.*, 2017*a* ▸). On the other hand, the numerical free-space propagation of the complex amplitude produced from the noisy detected fluence, followed by the TIE-Hom retrieval, simply returned the noisy detected image to its original state. This is confirmed by the clear similarity of Fig. 1 ▸(*f*) with Fig. 1 ▸(*b*). The latter behaviour can be considered ‘reasonable’, because equation (20) is an exact inverse of equation (4) which approximates the numerical Fresnel diffraction used to obtain Fig. 1 ▸(*e*) from Fig. 1 ▸(*b*). These qualitative observations indicate that the ‘true’ PBI imaging (as typically implemented in experiments), consisting of free-space propagation of a complex amplitude with subsequent addition of noise and PSF blurring, followed by the TIE-Hom retrieval, violates the NRU by reducing noise without deterioration of the spatial resolution (*cf*. the remarks on the noisy-channel coding theorem in Section 1). At the same time, the ‘numerical’ PBI imaging, consisting of free-space propagation of the monomorphous complex amplitude constructed from the noisy detected fluence in the object plane, followed by the TIE-Hom retrieval, conforms to the NRU, by increasing the SNR and spoiling the spatial resolution at the retrieval stage by the same amounts as the decrease in the SNR and improvement of the spatial resolution at the forward propagation simulation stage. Therefore, these simulations are qualitatively fully consistent with the theoretical considerations presented in the previous section.

We now proceed with quantitative analysis of the SNR and spatial resolution in the images shown in Fig. 1 ▸, using software implementation of equations (5)–(7) and (15). The following points explain our approach to this analysis and its results.

(1) All measurements of the SNR have been performed in the ‘flat’ region located in the top-right corner of the images. As expected, the SNR remained the same after the free-space propagation and it increased upon the TIE-Hom phase retrieval. The latter effect can be seen by comparing the measured values in cells c2 and d2 of Table 1 ▸, and, similarly, the values in cells e2 and f2.

(2) Regarding the measurements of spatial resolution, we have found that, for practical applications, it is more convenient to normalize the resolution slightly differently from the normalization used in equation (6). The following normalization leads to measured values of the spatial resolution which are close to the ones naturally expected from *a priori* knowledge about the imaging conditions, particularly in the context of experimental images considered later in the paper,
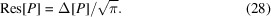
Note that equation (28) effectively corresponds to the width of a function defined as twice the 1D standard deviation. For example, for Gaussian PSFs, 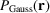 = 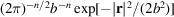, the variance is equal to *b*^2^ in 1D, 2*b*^2^ in 2D and 3*b*^2^ in 3D, and in all these cases equation (28) gives Res[*P*_Gauss_] = 2*b*. The corresponding resolution measurements in images from Fig. 1 are given in Table 1 ▸, with column 3 containing the measurements performed in the top-right (flat) region and those in column 5 containing the measurements performed in the top-left (patterned) region. The results in column 3 reflect only the effect of the convolution with the relevant filter functions in the ‘flat’ areas with Poisson noise. For example, the measured values of 257 µm and 261 µm in cells b3 and c3 of Table 1 ▸, respectively, agree well with the known width of the Gaussian PSF of the detector, with 2*b* = 250 µm. On the other hand, the values in column 5 contain also the contribution from the ‘intrinsic’ PSF of the pattern in the top-left corner, *i.e.* the Lorentzian filter with the FWHM of approximately 1054 µm. Note that the latter value is close to the measured value in cell b5 of Table 1 ▸.

(3) The TIE-Hom approximation to the free-space propagation in the near-Fresnel region is described by equation (19) with the filter function 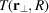 = 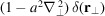, whose second integral moment is equal to −4*a*^2^. The improvement of the spatial resolution due to this filter function can be expressed as Δ^2^[*P*_1_] ≅ Δ^2^[*P*_0_] − 8π*a*^2^, where 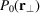 and 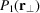 are the effective PSFs in the object and in the image planes, respectively. This implies that Res[*P*_1_] ≅ (Res^2^[*P*_0_] − 8*a*^2^)^1/2^. Under the conditions used in the present simulations, we obtain 8*a*^2^ = (2/π)γ*R*λ ≅ 636620 µm^2^. Accordingly, the improvement in the spatial resolution in the pattern in the top left corner of the image, as a result of free-space propagation, is expected to be from Res[*P*_0_] = 1066 µm (cell b5 in Table 1 ▸) to approximately Res[*P*_1_] = (1066^2^ µm^2^ − 636620 µm^2^)^1/2^ ≅ 707 µm. The latter number is close to the measured value of 716 µm in cell c5 of Table 1 ▸.

(4) Similarly to the previous point, the effect of the application of TIE-Hom retrieval on spatial resolution can be estimated via the addition of the second integral moment of the corresponding filter function, 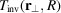

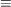

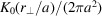
, which is equal to 4*a*^2^, to the second moment of *P*_1_. The resultant resolution is then equal to Res[*P*_0, retr_] = (Res^2^[*P*_1_] + Res^2^[*T*_inv_])^1/2^, where 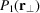 and 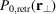 are the effective PSFs in the image plane and in the object plane after the TIE-Hom retrieval, respectively. Under the conditions of our simulations, we have Res^2^[*T*_inv_] = 8*a*^2^ ≅ 636620 µm^2^ and the measured value of Res[*P*_1_] = 716 µm is given in cell c5 of Table 1 ▸. Hence, the expected value of Res[*P*_0, retr_] is (716^2^ µm^2^ + 636620 µm^2^)^1/2^ ≅ 1072 µm, which is close to the measured value of 1068 µm in cell d5 of Table 1 ▸.

(5) For the parameters used in this simulation, we have γ = 100, *N*_F_ ≅ 19.6, and hence, according to equation (27), the expected gain factor *G*_2_ should be approximately *G*_2_ = (100/19.6)^1/2^ ≅ 2.26. This theoretically predicted gain factor agrees reasonably well with the ratio of the measured values of SNR in cells b2 and d2 of Table 1 ▸: 248/91 ≃ 2.73, and with the measured SNR/res ratios given in cells b6 and d6: 0.23/0.09 ≃ 2.56. These measured gain factor values can be compared with the results obtained after the simulated free-space propagation of the complex amplitude 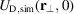 = 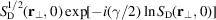, produced from the noisy blurred registered fluence in the detector plane, followed by the TIE-Hom retrieval. As indicated by the measured values in cells b2 and f2, as well as b6 and f6 of Table 1 ▸, we see that the gain factor in these simulations was exactly 1. This result is completely in line with the theoretical predictions given in the previous section.

### 5.2. Experimental PBI imaging

We also measured SNR and spatial resolution in experimental X-ray images collected at the Imaging and Medical beamline (IMBL) of the Australian Synchrotron. In the experiment, a plane monochromatic X-ray beam with energy of 32 keV was used, and the propagation distances were *R* = 15 cm (approximating the ‘contact’ image) and *R* = 600 cm (representing a typical PBI regime). Images were collected with a Xineos 3030HR flat-panel detector which had a pixel size of 99 µm × 99 µm and a PSF with Res ≃ 150 µm (Arhatari *et al.*, 2021 ▸). As the propagation distance from the ‘object’ to the ‘image’ planes was *R*′ = 585 cm and the wavelength was λ = 0.3875 Å, the corresponding minimal Fresnel number was *N*_F_ = π(Res)^2^/(*R*λ) ≅ 312. The imaged object was an excised mastectomy sample with an intermediate grade ductal carcinoma. This breast tissue sample could be considered approximately monomorphous with γ ≡ (δ_gland_ − δ_fat_)/(β_gland_ − β_fat_) = 869 at the specified X-ray energy (Gureyev *et al.*, 2019 ▸). Consequently, according to equation (27), the gain factor corresponding to PBI with *R*′ = 585 cm, relative to the ‘contact’ images, was expected to be approximately *G*_2_ = (γ/*N*_F_)^1/2^ ≅ 1.67.

**Figure 2:**
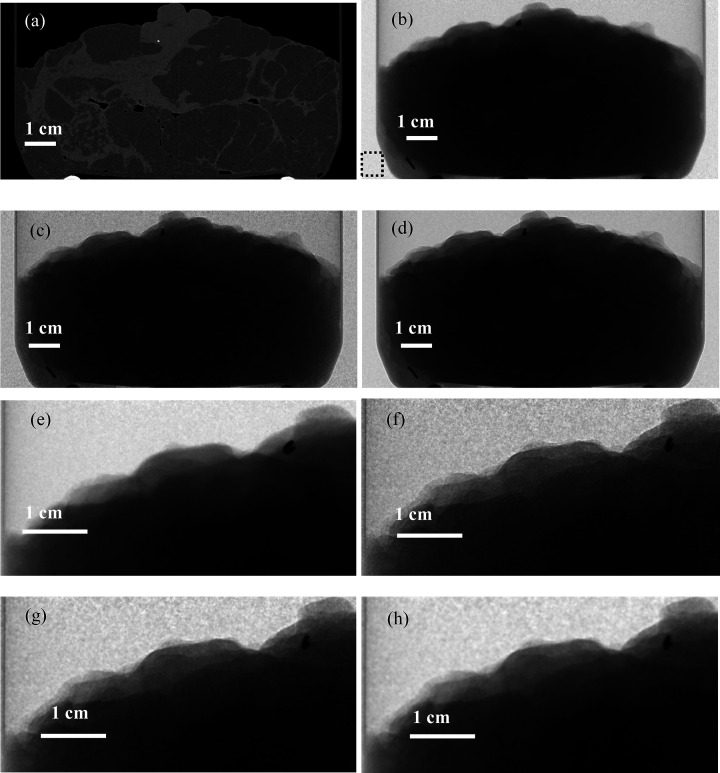
Images of a mastectomy sample collected using plane monochromatic X-rays with an energy of 32 keV. (*a*) Reconstructed axial CT slice through the middle of the sample. (*b*) PBI projection at the sample-to-detector distance of 19 cm and 3.33 µGy dose. The dashed square in the lower left corner outlines the region inside which the SNR measurements were performed in all the images. (*c*) PBI projection at the sample-to-detector distance of 600 cm and 0.67 µGy dose. (*d*) PBI projection at the sample-to-detector distance of 600 cm and 4 µGy dose. (*e*) Zoomed sub-image of (*b*). (*f*) Zoomed sub-image of (*c*). (*g*) Zoomed sub-image of the TIE-Hom reconstruction with γ = 275 from the image shown in (*c*). (*h*) Zoomed sub-image of the TIE-Hom reconstruction with γ = 869 from the image shown (*c*).

Fig. 2 ▸(*a*) shows a CT-reconstructed central slice through the mastectomy sample. This figure is included only to illustrate the general internal structure of the sample, which cannot be readily discerned in the subsequent 2D projection images. Fig. 2 ▸(*b*) contains an experimental PBI projection at the sample-to-detector distance of 19 cm and 3.33 µGy dose. Due to the small propagation distance, this image represents a good approximation for a conventional ‘contact’ CT projection. Fig. 2 ▸(*c*) shows a PBI projection at the sample-to-detector distance of 600 cm and 0.67 µGy dose, while Fig. 2 ▸(*d*) depicts a PBI projection at the same propagation distance, but at a higher dose of 4 µGy. The latter two figures demonstrate, in particular, that the spatial resolution in the PBI projections is noticeably higher than in the ‘contact’ projection shown in Fig. 2 ▸(*b*). This is particularly easy to see by comparing the image in Fig. 2 ▸(*e*) with that in Fig. 2 ▸(*f*), these images containing the same zoomed sub-region from Figs. 2 ▸(*b*) and 2 ▸(*c*), respectively. Finally, Figs. 2 ▸(*g*) and 2 ▸(*h*) contain the same zoomed sub-region after the TIE-Hom reconstruction from the image shown in Fig.2(*c*), according to equation (20) with γ = 275 and γ = 869, respectively. These last two figures clearly show an improvement in the SNR, compared with Fig. 2 ▸(*f*), at the expense of some deterioration in the spatial resolution, both effects appearing due to the low-pass filtering in the form of the TIE-Hom retrieval in accordance with equations (20)–(22).

We now proceed with the results of quantitative measurements of SNR and spatial resolution in the experimental images shown in Fig. 2 ▸. The SNR was measured in accordance with equations (7) and (16) within the uniform region of the images corresponding to the dotted square shown in Fig. 2(*b*) ▸. The spatial resolution was evaluated on the basis of estimation of the edge-spread function (Cunningham & Fenster, 1987 ▸; Gureyev *et al.*, 2008 ▸) at the top edge of the sample. This edge-based spatial resolution was denoted Res′ to distinguish it from the MTF-based resolution measurements used in Section 5.1. We had to resort to the edge-based resolution measurements here because, as explained in Section 5.1 above, it is impossible to detect changes in the MTF-based spatial resolution in PBI imaging without the presence of a suitable high-resolution high-contrast structure in the sample, *e.g.* similar to the one embedded in the top-left corner of the simulated sample used in Section 5.1. The edge-based resolution measured in the PBI projections collected at *R* = 19 cm and *R* = 600 cm was, respectively, 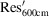 = 214 µm and 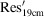 = 120 µm (see cells b3 and c3 in Table 2 ▸). These numbers were generally consistent with the known detector resolution Res_det_ ≃ 150 µm in view of: (i) an expected contribution of the inherent width of the sample edge to the measured resolution, and (ii) an expected improvement in the spatial resolution as a result of the free-space propagation. The measured SNR was essentially the same in the images collected at *R* = 19 cm and *R* = 600 cm without the TIE-Hom retrieval, when the actual radiation doses were taken into account. Indeed, for example, the measured value SNR_3.33µGy_/SNR_0.67µGy_ = 52/23 = 2.26 (see cells b2 and c2 in Table 2 ▸) was close to the known ratio of the doses (3.33/0.67)^1/2^ = 2.23. Most importantly, the measured values of the intrinsic imaging quality characteristic, *Q*_*S*_, and the gain factor, *G*_2_, were consistent with the theory presented in Section 4 above. As explained after equation (27), for a fixed sample the absorbed radiation dose is proportional to the incident fluence. Therefore, the values in column 4 of Table 2 ▸ are proportional to *Q*_*S*_ and their ratios are equal to the gain factor. It is easy to see in cells c4–h4 of Table 2 ▸ that the measured values of *Q*_*S*_ were basically the same for all the PBI images at *R* = 600 cm, regardless of the dose or the application of TIE-Hom retrieval with different values of the parameter γ. This confirms the theoretically predicted independence of the intrinsic imaging quality of the dose and of the TIE-Hom retrieval, since the latter is an example of linear filtering which always leaves *Q*_*S*_ unchanged. On the other hand, the ratio of the values in cells c4 and b4 of Table 2 ▸ is equal to 0.23/013 ≃ 1.77, which is close to the theoretical value of *G*_2_ = (γ/*N*_F_)^1/2^ ≅ 1.67 calculated above.

More detailed and comprehensive analysis of the behaviour of SNR and spatial resolution in 2D and 3D (CT) experimental PBI images will be presented in the second part of this work in a later publication.

## 6. Conclusions

It follows from previous publications (Paganin, 2006 ▸; Nesterets & Gureyev, 2014 ▸; Gureyev *et al.*, 2017*a* ▸) and the results presented above that the performance of Paganin’s method for PBI of monomorphous objects is determined by just two key dimensionless parameters: the Fresnel number, *N*_F_ = Δ^2^/(*R*λ) (where Δ is the spatial resolution of the detector, *R* is object-to-detector distance and λ is the radiation wavelength) and the ratio γ = δ/β of the real decrement to the imaginary part of the refractive index of the imaged object. In particular, the gain in SNR, or, equivalently, in the SNR-to-resolution ratio, in 2D free-space propagation followed by the TIE-Hom retrieval, is determined by the ratio of γ and *N*_F_: *G*_2_ = (γ/*N*_F_)^1/2^, see equations (26) and (27). Note that this gain factor depends on the dimensionality of the images: in 1D it becomes *G*_1_ ≅ (γ/*N*_F_)^1/4^ and in 3D *G*_3_ ≅ (γ/*N*_F_)^3/4^ (see the second part of the present paper) or *G*_3_ ≅ γ/*N*_F_ (Nesterets & Gureyev, 2014 ▸), depending on the exact definition of the Fresnel number. The improvement of the spatial resolution upon free-space propagation is determined by the second integral moment, −4*a*^2^ = −γ*R*λ/π = −Δ^2^γ/(π*N*_F_), of the forward TIE-Hom filter (deconvolution) function, 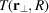 = 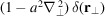, see equation (19). This implies that the improvement in the spatial resolution due to free-space propagation is essentially determined by the same parameter *G*_2_. More detailed and accurate theoretical estimates of the gain factor is given by Nesterets & Gureyev (2014 ▸).

The gain factor *G*_*n*_ quantifies the ‘degree’ of violation of NRU in PBI and, hence, the effectiveness of Paganin’s method. In view of the arguments presented in Section 4 above, the latter effectiveness can be understood as the advantage that a ‘hardware’ implementation of PBI can achieve over ‘software’ implementations in the form of computer processing of conventional absorption-based images collected at the same radiation dose. Since the Fresnel number in PBI typically has to be larger than unity in order to satisfy the validity conditions of the method, γ needs to be even larger in order for the gain factor to be larger than one, *i.e.* for the method to work effectively. Fortunately, γ = δ/β is typically of the order of 10^3^ to 10^4^ for soft biological tissues (composed of light chemical elements) when they are imaged using hard X-rays with wavelengths shorter than 1 Å, which correspond to X-ray energies higher than approximately 12 keV. Importantly, at such X-ray energies, many types of soft biological tissues can be considered approximately monomorphous. For this reason, hard X-ray PBI in general and Paganin’s method in particular have become popular in biomedical applications in recent years (Wilkins *et al.*, 2014 ▸; Taba *et al.*, 2018 ▸; Endrizzi, 2018 ▸; Quenot *et al.*, 2022 ▸). Note that a popular practical strategy associated with Paganin’s method is to use smaller values, γ′ < γ, in the TIE-Hom retrieval for processing of experimental images (Gureyev *et al.*, 2019 ▸). This typically leads to sharper reconstructed images due to incomplete compensation of the edge-enhancement effect of the coherent free-space propagation. This improved spatial resolution tends to lead to higher subjective radiological image quality scores compared with images reconstructed with the ‘true’ value of γ = δ/β (Taba *et al.*, 2019 ▸). Importantly, as shown in the present paper, such changes in the value of γ′, and hence in the parameter *a*′^2^ = γ′*R*λ/(4π), in the TIE-Hom retrieval algorithm, equation (20), leave the gain factor *G*_2_ unchanged. This invariance of the gain factor with respect to γ′ is a direct consequence of the noise-resolution duality principle (Gureyev *et al.*, 2014 ▸), according to which any increases in the spatial resolution obtained as a result of using smaller values of γ′ in the TIE-Hom retrieval are always accompanied by lowering of SNR in the images.

The fact that the PBI gain factor can be much larger in 3D imaging (Nesterets & Gureyev, 2014 ▸; Kitchen *et al.*, 2017 ▸) than in planar imaging makes PCT a particularly attractive approach for 3D imaging applications. The method is currently being adopted for medical imaging of live humans (Gureyev *et al.*, 2019 ▸; Brombal *et al.*, 2019 ▸; Arhatari *et al.*, 2021 ▸). In this context, it is important to analyse the details of SNR improvement in PCT under practical conditions which require minimization of both the radiation dose and the exposure time. Such analysis can help researchers and engineers to optimize future medical instruments for PCT imaging using synchrotron radiation and laboratory X-ray sources. This serves as a key motivation for the detailed quantitative study presented here and in the second part of the present paper, which further develops and applies the theoretical framework described in the present paper to experimental PCT images, with particular emphasis on the role of photon-counting detectors in such applications (Brombal *et al.*, 2018 ▸, 2019 ▸).

## Figures and Tables

**Table 1:** SNR and spatial resolution [‘Res’, equation (28)) measured in images shown in Figs. 1 ▸(*b*)–1(*f*) (row indices in the table correspond to the panes of Fig. 1 ▸) Spatial resolution results given in columns 3 and 5 were based on the measurement of the width of the central peak of the MTF. The results in columns 2–4 were obtained in the flat area in the top-right corner of the images, while the results in column 5 were obtained in the patterned top-left corner of the images. The results in column 6 were obtained by dividing the values in column 2 by the value in the same row of column 5.

	1	2	3	4	5	6
	Image	SNR	Res (µm)	SNR/Res (µm^−1^)	Res′ (µm)	SNR/Res′ (µm^−1^)
b	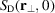	**91**	257	0.36	1066	0.09
c	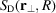	93	261	0.36	**716**	0.13
d	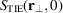	**248**	513	0.48	**1068**	**0.23**
e	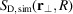	11	165	0.07	372	0.03
f	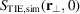	92	257	0.36	1066	0.09

**Table 2:** SNR and spatial resolution measured in the experimental PBI images of a mastectomy sample collected with plane monochromatic X-rays with *E* = 32 keV

	1	2	3	4
	Projection images	SNR	Res′ (µm)	SNR/Res′/Dose^1/2^ (µm^−1^ µGy^−1/2^)
b	*R* = 19 cm, dose = 3.33 µGy	52	214	0.13
c	*R* = 600 cm, dose = 0.67 µGy	23	120	0.23
d	*R* = 600 cm, dose = 4.00 µGy	55	119	0.23
e	TIE-Hom retrieval with γ = 275, *R* = 600 cm, dose = 0.67 µGy	34	193	0.22
f	TIE-Hom retrieval with γ = 275, *R* = 600 cm, dose = 4.00 µGy	82	183	0.22
g	TIE-Hom retrieval with γ = 869, *R* = 600 cm, dose = 0.67 µGy	47	231	0.25
h	TIE-Hom retrieval with γ = 869, *R* = 600 cm, dose = 4.00 µGy	109	224	0.24

## References

[bb1] Abramowitz, M. & Stegun, I. A. (1972). *Handbook of Mathematical Functions with Formulas, Graphs, and Mathematical Tables.* New York: Dover.

[bb2] Ando, M. & Hosoya, S. (1972). *Proceedings of the Sixth International Conference on X-ray Optics and Microanalysis*, edited by G. Shinoda, K. Kohra, & T. Ichinokawa, pp. 63–68. University of Tokyo Press.

[bb3] Arhatari, B. D., Stevenson, A. W., Abbey, B., Nesterets, Y. I., Maksimenko, A., Hall, C. J., Thompson, D., Mayo, S. C., Fiala, T., Quiney, H. M., Taba, S. T., Lewis, S. J., Brennan, P. C., Dimmock, M., Häusermann, D. & Gureyev, T. E. (2021). *Appl. Sci.* **11**, 4120.

[bb4] Barrett, H. H. & Myers, K. J. (2004). *Foundations of Image Science.* New York: John Wiley & Sons.

[bb5] Bohm, D. (1952*a*). *Phys. Rev.* **85**, 166–179.

[bb6] Bohm, D. (1952*b*). *Phys. Rev.* **85**, 180–193.

[bb7] Bonse, U. & Hart, M. (1965). *Appl. Phys. Lett.* **6**, 155–156.

[bb8] Born, M. & Wolf, E. (1999). *Principles of Optics: Electromagnetic Theory of Propagation, Interference and Diffraction of Light*, 7th ed. Cambridge University Press.

[bb9] Brombal, L., Arfelli, F., Delogu, P., Donato, S., Mettivier, G., Michielsen, K., Oliva, P., Taibi, A., Sechopoulos, I., Longo, R. & Fedon, C. (2019). *Sci. Rep.* **9**, 17778. 10.1038/s41598-019-54131-z PMC6882794 31780707

[bb10] Brombal, L., Golosio, B., Arfelli, F., Bonazza, D., Contillo, A., Delogu, P., Donato, S., Mettivier, G., Oliva, P., Rigon, L., Taibi, A., Tromba, G., Zanconati, F. & Longo, R. (2018). *J. Med. Imag.* **6**, 031402. 10.1117/1.JMI.6.3.031402 PMC6257100 30525064

[bb11] Cloetens, P., Barrett, R., Baruchel, J., Guigay, J. P. & Schlenker, M. (1996). *J. Phys. D Appl. Phys.* **29**, 133–146.

[bb12] Cloetens, P., Pateyron-Salomé, M., Buffière, J. Y., Peix, G., Baruchel, J., Peyrin, F. & Schlenker, M. (1997). *J. Appl. Phys.* **81**, 5878–5886.

[bb13] Cunningham, I. A. & Fenster, A. (1987). *Med. Phys.* **14**, 533–537. 10.1118/1.596064 3626992

[bb14] Davis, T. J., Gao, D., Gureyev, T. E., Stevenson, A. W. & Wilkins, S. W. (1995). *Nature*, **373**, 595–598.

[bb16] Dolby, R. M. (1968). *Audio*, **55**, 19–22.

[bb17] Dreier, I., Ehm, W., Gneiting, T. & Richards, D. (2001). *Math. Nachr.* **228**, 109–122.

[bb18] Endrizzi, M. (2018). *Nucl. Instrum. Methods Phys. Res. A*, **878**, 88–98.

[bb19] Folland, G. B. & Sitaram, A. (1997). *J. Fourier Anal. Appl.* **3**, 207–238.

[bb20] Förster, E., Goetz, K. & Zaumseil, P. (1980). *Cryst. Res. Technol.* **15**, 937–945.

[bb21] García-Moreno, F., Kamm, P. H., Neu, T. R., Bülk, F., Mokso, R., Schlepütz, C. M., Stampanoni, M. & Banhart, J. (2019). *Nat. Commun.* **10**, 3762. 10.1038/s41467-019-11521-1 PMC6704127 31434878

[bb22] García–Moreno, F., Kamm, P. H., Neu, T. R., Bülk, F., Noack, M. A., Wegener, M., von der Eltz, N., Schlepütz, C. M., Stampanoni, M. & Banhart, J. (2021). *Adv. Mater.* **33**, 2104659. 10.1002/adma.202104659 PMC11468671 34558111

[bb23] Goodman, P. (2000). *Statistical Optics.* New York: Wiley.

[bb24] Gureyev, T. E., de Hoog, F. R., Nesterets, Y. & Paganin, D. M. (2015). *ANZIAM J.* **56**, C1–C15.

[bb25] Gureyev, T. E., Kozlov, A., Paganin, D. M., Nesterets, Y. I., De Hoog, F. & Quiney, H. M. (2017*b*). *J. Opt. Soc. Am. A*, **34**, 1577–1584. 10.1364/JOSAA.34.001577 29036160

[bb26] Gureyev, T. E., Kozlov, A. Ya. I., Paganin, D. M., Nesterets, Y. I. & Quiney, H. M. (2020). *Sci. Rep.* **10**, 7890. 10.1038/s41598-020-64539-7 PMC7217923 32398680

[bb27] Gureyev, T. E., Nesterets, Y. I. & de Hoog, F. (2016). *Opt. Express*, **24**, 17168–17182. 10.1364/OE.24.017168 27464167

[bb28] Gureyev, T. E., Nesterets, Y. I., de Hoog, F., Schmalz, G., Mayo, S. C., Mohammadi, S. & Tromba, G. (2014). *Opt. Express*, **22**, 9087–9094. 10.1364/OE.22.009087 24787797

[bb29] Gureyev, T. E., Nesterets, Y. I., Kozlov, A., Paganin, D. M. & Quiney, H. M. (2017*a*). *J. Opt. Soc. Am. A*, **34**, 2251–2260. 10.1364/JOSAA.34.002251 29240102

[bb30] Gureyev, T. E., Nesterets, Y. I., Paganin, D. M., Pogany, A. & Wilkins, S. W. (2006). *Opt. Commun.* **259**, 569–580.

[bb31] Gureyev, T. E., Nesterets, Y. I., Stevenson, A. W., Miller, P. R., Pogany, A. & Wilkins, S. W. (2008). *Opt. Expr.* **16**, 32233241. 10.1364/oe.16.003223 18542410

[bb32] Gureyev, T. E., Nesterets, Y. I., Stevenson, A. W. & Wilkins, S. W. (2003). *Appl. Opt.* **42**, 6488–6494. 10.1364/ao.42.006488 14650491

[bb33] Gureyev, T. E., Stevenson, A. W., Nesterets, Y. I. & Wilkins, S. W. (2004). *Opt. Commun.* **240**, 81–88.

[bb34] Gureyev, T. E. & Wilkins, S. W. (1997). *Nouv Cim D*, **19**, 545–552.

[bb35] Gureyev, T. E. Ya. I., Nesterets, Y. I., Baran, P. M., Taba, S. T., Mayo, S. C., Thompson, D., Arhatari, B., Mihocic, A., Abbey, B., Lockie, D., Fox, J., Kumar, B., Prodanovic, Z., Hausermann, D., Maksimenko, A., Hall, C., Peele, A. G., Dimmock, M., Pavlov, K. M., Cholewa, M., Lewis, S., Tromba, G., Quiney, H. M. & Brennan, P. C. (2019). *Med. Phys.* **46**, 5478–5487. 10.1002/mp.13842 31574166

[bb15] Hoog, F. de, Schmalz, G. & Gureyev, T. E. (2014). *Appl. Math. Lett.* **38**, 84–86.

[bb36] Ingal, V. N. & Beliaevskaya, E. A. (1995). *J. Phys. D Appl. Phys.* **28**, 2314–2317.

[bb37] Kitchen, M. J., Buckley, G. A., Gureyev, T. E., Wallace, M. J., Andres-Thio, N., Uesugi, K., Yagi, N. & Hooper, S. B. (2017). *Sci. Rep.* **7**, 15953. 10.1038/s41598-017-16264-x PMC5698457 29162913

[bb38] MacKay, D. J. C. (2003). *Information Theory, Inference, and Learning Algorithms.* Cambridge University Press.

[bb39] Mandel, L. & Wolf, E. (1962). *Proc. Phys. Soc.* **80**, 894–897.

[bb40] Mandel, L. & Wolf, E. (1995). *Optical Coherence and Quantum Optics.* Cambridge University Press.

[bb41] Mizutani, R., Saiga, R., Takekoshi, S., Inomoto, C., Nakamura, N., Itokawa, M., Arai, M., Oshima, K., Takeuchi, A., Uesugi, K., Terada, Y. & Suzuki, Y. (2016). *J. Microsc.* **261**, 57–66. 10.1111/jmi.12315 26444300

[bb42] Momose, A. (1995). *Nucl. Instrum. Methods Phys. Res. A*, **352**, 622–628.

[bb43] Nesterets, Y. I. & Gureyev, T. E. (2014). *J. Phys. D Appl. Phys.* **47**, 105402.

[bb44] Nicolic, H. (2005). *Found. Phys. Lett.* **18**, 549–561.

[bb45] Nugent, K. A., Gureyev, T. E., Cookson, D. F., Paganin, D. & Barnea, Z. (1996). *Phys. Rev. Lett.* **77**, 2961–2964. 10.1103/PhysRevLett.77.2961 10062096

[bb46] Paganin, D., Mayo, S. C., Gureyev, T. E., Miller, P. R. & Wilkins, S. W. (2002). *J. Microsc.* **206**, 33–40. 10.1046/j.1365-2818.2002.01010.x 12000561

[bb47] Paganin, D. M. (2006). *Coherent X-ray Optics.* Oxford University Press.

[bb48] Petersen, T. C., Paganin, D. M., Weyland, M., Simula, T. P., Eastwood, S. A. & Morgan, M. J. (2014). *Phys. Rev. A*, **89**, 063801. 10.1103/PhysRevLett.110.033901 23373924

[bb49] Quenot, L., Bohic, S. & Brun, E. (2022). *Appl. Sci.* **12**, 9539.

[bb50] Raven, C., Snigirev, A., Snigireva, I., Spanne, P., Souvorov, A. & Kohn, V. (1996). *Appl. Phys. Lett.* **69**, 1826–1828.

[bb51] Sakurai, J. J. (1967). *Advanced Quantum Mechanics.* Massachusetts: Addison-Wesley.

[bb52] Shannon, C. E. (1948*a*). *Bell Syst. Tech. J.* **27**, 379–423.

[bb53] Shannon, C. E. (1948*b*). *Bell Syst. Tech. J.* **27**, 623–656.

[bb54] Snigirev, A., Snigireva, I., Kohn, V., Kuznetsov, S. & Schelokov, I. (1995). *Rev. Sci. Instrum.* **66**, 5486–5492.

[bb56] Taba, S. T., Gureyev, T. E., Alakhras, M., Lewis, S., Lockie, D. & Brennan, P. (2018). *Am. J. Roentgen.* **211**, 131–145. 10.2214/AJR.17.19179 29792739

[bb55] Tavakoli Taba, S., Baran, P., Lewis, S., Heard, R., Pacile, S., Nesterets, Y. I., Mayo, S. C., Dullin, C., Dreossi, D., Arfelli, F., Thompson, D., McCormack, M., Alakhras, M., Brun, F., Pinamonti, M., Nickson, C., Hall, C., Zanconati, F., Lockie, D., Quiney, H. M., Tromba, G., Gureyev, T. E. & Brennan, P. C. (2019). *Acad. Radiol.* **26**, e79–e89. 10.1016/j.acra.2018.07.008 30149975

[bb57] Teague, M. R. (1983). *J. Opt. Soc. Am.* **73**, 1434–1441.

[bb58] Vladimirov, V. S. (2002). *Methods of the Theory of Generalized Functions.* CRC Press.

[bb59] Wilkins, S. W., Gureyev, T. E., Gao, D., Pogany, A. & Stevenson, A. W. (1996). *Nature*, **384**, 335–338.

[bb60] Wilkins, S. W., Nesterets, Y. I., Gureyev, T. E., Mayo, S. C., Pogany, A. & Stevenson, A. W. (2014). *Philos. Trans. R. Soc. A*, **372**, 20130021. 10.1098/rsta.2013.0021 24470408

